# Influence of Dental Pain and Tooth Sensitivity on the Intention to Quit Smoking among Schoolchildren

**DOI:** 10.1155/2020/8823146

**Published:** 2020-07-09

**Authors:** Muhammad Nazir, Hassan AlAbdullah, Muhanad Alhareky, Asim Al-Ansari, Jehan Alhumaid

**Affiliations:** ^1^Department of Preventive Dental Sciences, College of Dentistry, Imam Abdulrahman Bin Faisal University, P.O. Box 1982, Dammam 31441, Saudi Arabia; ^2^College of Dentistry, Imam Abdulrahman Bin Faisal University, P.O. Box 1982, Dammam 31441, Saudi Arabia

## Abstract

**Objective:**

To evaluate the association between oral health problems and sociodemographic factors with the intention to quit smoking and join the tobacco cessation program among schoolchildren.

**Materials and Methods:**

This cross-sectional study included a sample of 10–12 grade male students from public high schools in different cities of the Eastern province of Saudi Arabia. Schoolchildren responded to a pilot-tested questionnaire about self-reported oral health problems and the intention to quit smoking. Bivariate and multivariate logistic regression analyses were performed.

**Results:**

Out of 587 schoolchildren, 199 were smokers with a smoking prevalence of 33.9%. Significantly higher proportions of smokers than nonsmokers had tooth sensitivity (*P* = 0.029) and dryness of the mouth (*P* = 0.001). Most smokers (75.9%) had a family history of smoking, and 51.8% started smoking within the last two years. Tooth sensitivity (56.3%), dental cavities (52.8%), and dental pain (44.7%) were highly prevalent among smokers. About one-third of smokers expressed their intention to quit smoking (38.2%) and join a smoking cessation program (33.7%). Tooth sensitivity (OR = 3.7, *P* = 0.004) and dental pain (OR = 2.84, *P* = 0.014) were significantly associated with quitting smoking. In addition, smokers with tooth sensitivity (OR = 3.22, *P* = 0.007) had higher odds of joining a smoking cessation program than those without tooth sensitivity. The smokers who started smoking within the last two years (OR = 3.97, *P* = 0.002) were more likely to quit smoking than those who initiated smoking for more than two years.

**Conclusion:**

Oral health problems were highly prevalent among smokers. Tooth sensitivity was significantly associated with quitting smoking and joining a cessation program in children. The awareness about the adverse effects of smoking on oral health should be part of regional, national, and global tobacco prevention policies and programs.

## 1. Introduction

According to the World Health Organization (WHO) estimates, there were more than 1.1 billion smokers in the world in 2015 [1]. Smoking causes cancer, respiratory disease, heart disease, and stroke, which lead to disability and death [2]. In addition, tobacco use is associated with an increased risk of mortality from communicable diseases [3]. Globally, six million people die because of tobacco use each year and many deaths are premature. These mortality estimates are projected to reach more than eight million per year in 2030 [3]. Tobacco use causes the death of one person every six seconds [4]. Moreover, the economic burden of smoking-related diseases accounts for 1.5%–6.8% of national healthcare costs of a country [5]. In addition to the high prevalence of smoking, oral problems are common resulting in most dental visits due to pain, and routine dental care is scant among adolescents [6, 7].

Adolescence is the most rapid developmental period of humans, and adolescents are vulnerable to emotional, social, health, development, and vocational issues because of changes in them and/or their environment [8]. Hence, adolescents are at increased risk of suicidal tendencies, self-injurious behaviors, anxiety, substance consumption, family problems, and mental issues [9]. Globally, tobacco consumption is a major public health problem among adolescents [9]. It has been reported that 1 in 10 adolescents use tobacco worldwide [10]. In Saudi Arabia, the studies conducted in different cities reported prevalence estimates of smoking (20.2%–37%) among male adolescents [11–14]. According to the U.S. Surgeon General's report, more than 80% of adult smokers initiate tobacco consumption before the age of 18 years, and if adolescents do not smoke during adolescence, then they are very unlikely to smoke in adulthood [2].

It is known that the health-related adverse effects of tobacco use are a major reason for quitting smoking [15–17]. The motivation to quit smoking is also influenced by medical advice and awareness about the benefits of quitting [16]. However, smokers with nicotine dependence are less likely to quit or have the intention to quit smoking [18]. A previous study found an association between the history of lung disease and the intention to quit smoking among Swiss smokers [19]. Similarly, Iranian smokers who were diagnosed with tuberculosis were willing to quit smoking [20]. Among Koreans, the intention of quitting was significantly associated with tooth brushing after lunch [21]. In Saudi Arabia, a study found that schoolchildren who were aware of the negative effects of smoking were three times more likely to quit smoking than those who lack such awareness [22].

The intention to quit smoking is important for successful smoking cessation [21, 23]. Though quitting smoking is difficult primarily due to nicotine dependence and withdrawal symptoms [22, 24]. However, schoolchildren (15–19 years) who are aware of the adverse consequences of smoking on oral health are less likely to smoke [25]. High occurrence of smoking and its oral and systemic adverse effects calls for an investigation on quitting smoking. The benefits of behavioral modification during adolescence can be achieved by quitting smoking to improve the health and wellbeing of schoolchildren [26]. The understanding of the factors associated with the intention of quitting is critical to preventive initiatives aimed at controlling the epidemic of smoking among adolescents. Therefore, the study aimed to evaluate the factors that influence schoolchildren's intention to quit smoking and join a smoking cessation program.

## 2. Materials and Methods

### 2.1. Study Participants

This cross-sectional study (EA#2018009) was conducted on 10–12 grade male schoolchildren. They were recruited from public high schools in Dammam, AlKhobar, and Dhahran cities in the Eastern province of Saudi Arabia. These interconnected cities represent the third biggest metropolitan area with a population of 0.77 million in the country. The sample calculation involved estimating student populations in these cities, confidence limit (±3), design effect, and an estimated proportion of subjects with outcome (20.1%). This calculation created a sample of 661 schoolchildren. Seven schools in Dammam, AlKhobar, and Dhahran were randomly selected. The administrators of selected schools granted permission to conduct the study. In each selected school, eligible students were provided with information about the purpose and objective of the study. They were also informed that their participation is voluntary, and they have the right to withdraw from the study without negative consequences. The schoolchildren who were willing to participate in the study provided their written informed consent. However, parents/legal guardians provided written consent for the children who were under the age of 16 years. The participants were provided with explanations if they were unable to clearly understand the questions asked in the instrument. To maintain ethical standards in the study, the participants were not segregated as smokers and nonsmokers during data collection.

### 2.2. Instrument

The instrument used to collect data had three sections (see instrument attached as a supplementary file). The first section inquired respondents about sociodemographic information such as age, academic grades in the previous year, monthly family income, education of father and mother, initiation of smoking, and the family history of smoking. The second section of the instrument included items related to self-reported oral health problems. The respondents were asked to provide their responses about bleeding gums in the past month, dental pain in the past month, tooth sensitivity in the past month, dental cavities, dryness of the mouth, oral malodor, routine dental check-up, and satisfaction with dental appearance. The last section of the instrument was about participants' intention to quit smoking in the next six months and join a smoking cessation program. The instrument was developed in English language and discussed among researchers several times to ensure its content and face validity. Later, it was translated into Arabic language following the recommendations of the World Health Organization mentioned in the “Process of translation and adaptation of instrument” [27]. Finally, pretesting of the questionnaire was conducted on a group of 30 schoolchildren who were not included in the study.

### 2.3. Data Analysis

Data were analyzed using SPSS software (IBM SPSS Statistics for Windows, Version 22.0. Armonk, NY : IBM Corp). Descriptive statistics included frequencies and percentages of study variables. The chi-square test was performed to compare self-reported oral health problems between smokers and nonsmokers. Later, data of smokers were used to observe the influence of independent variables of the study such as initiation of smoking, the family history of smoking, bleeding gums, dental cavities, dental pain, and tooth sensitivity on dependent variables of the study (intention to quit smoking and intention to join smoking cessation program). Bivariate and multivariate logistic regression analyses were performed to explore an association between independent and dependents variables of the study. Furthermore, multivariate logistic regression backward stepwise final models were developed because of their best predictive power. A *P* value of <0.05 was considered statistically significant.

## 3. Results

**Figure 1:**
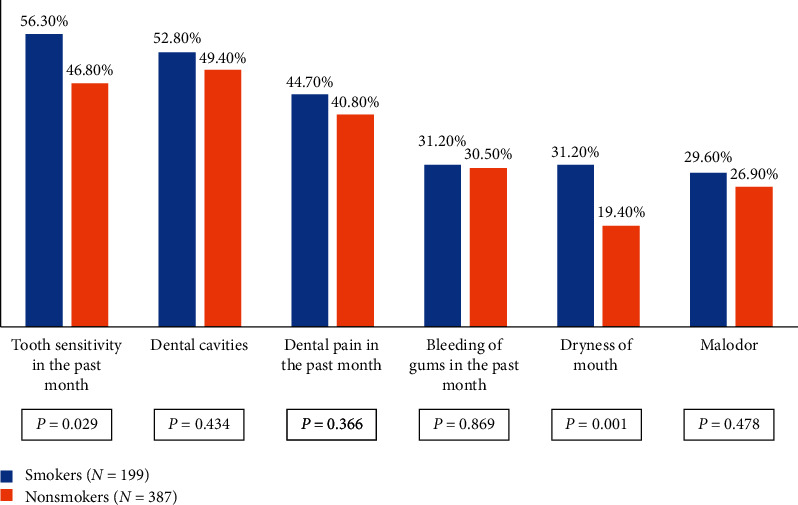
Comparison of self-reported oral problems between smoker and nonsmoker students.

Five hundred eighty-seven schoolchildren returned completed questionnaires (587 out of 661 yielded a response rate = 88.8). There were 199 smokers in the study with a smoking prevalence of 33.9%.  Figure 1 shows the comparison of self-reported oral problems between smokers and nonsmokers. Significantly higher proportions of smokers than nonsmokers had tooth sensitivity (*P* = 0.029) and dryness of the mouth (*P* = 0.001). However, the study found no statistically significant differences in the proportions of smokers and nonsmokers with regards to dental caries (*P* = 0.434), dental pain (*P* = 0.366), gum bleeding (*P* = 0.869), and malodor (*P* = 0.478).

Among smokers, 51.8% started smoking ≤ two years, and 75.9% had a family history of smoking. Tooth sensitivity (56.3%), dental cavities (52.8%), and dental pain (44.7%) were the three most commonly reported oral problems by the smokers. When asked about quitting smoking, 38.2% of smokers expressed their intention to quit smoking, and 33.7% were willing to join the smoking cessation program. Descriptive statistics are summarized in Table 1.

Table 2 demonstrates the results of bivariate and multivariate analyses. In bivariate analysis, the schoolchildren who started smoking in ≤two years were 2.54 times more likely to quit smoking than those who initiated smoking for more than two years (*P* = 0.002). Similarly, the schoolchildren with dental pain (OR = 2.61) and tooth sensitivity (OR = 2.27) were twice more likely to express their intention to quit. Other variables with statistically significant bivariate associations with intention to quit smoking included oral malodor (OR = 2.11) and university education of mother (OR = 2.1). After adjusting for other variables in multivariate logistic regression analyses, the initiation of smoking ≤2 years (OR = 3.69, *P* = 0.006), dental pain (OR = 3.29, *P* = 0.011), and tooth sensitivity (OR = 4.16, *P* = 0.007) were associated with the intention to quit smoking.

The multivariate logistic regression final model was created using a backward LR method. The results of the final model indicated that schoolchildren who experienced dental pain had significantly higher odds (OR = 2.84, *P* = 0.014) of quitting smoking than those who did not report dental pain. Similarly, the schoolchildren with tooth sensitivity were 3.7 times more likely to intend to quit smoking than those without tooth sensitivity (*P* = 0.004). In addition, the schoolchildren with university-educated mothers (OR = 2.76, *P*=0.04) and those with smoking initiation in ≤2 years (OR = 3.97, *P*=0.002) were significantly more likely to quit smoking (Table 3).

The results of the association of the factors with the intention to join a smoking cessation program are shown in Table 4. Bivariate analysis showed that the schoolchildren with dental pain (OR = 2.28, *P*=0.006) and tooth sensitivity (OR = 3.32, *P* ≤ 0.001) during the past month had significantly higher odds of joining a smoking cessation program. However, multivariate logistic regression analysis shows that tooth sensitivity during the past month was significantly associated with the higher likelihood (OR = 3.02, *P*=0.026) of joining a smoking cessation program.

The multivariate logistic regression backward stepwise final model demonstrated that schoolchildren with tooth sensitivity in the past month were 3.22 times more likely to join a smoking cessation program than those without tooth sensitivity (*P* = 0.007) (Table 5).

## 4. Discussion

The present study evaluated data about dental and sociodemographic factors associated with quitting smoking in schoolchildren in Saudi Arabia. The valuable information inferred from this study can be used by healthcare providers to promote quitting smoking and refer smokers to tobacco cessation programs. Similarly, the decision-makers in the health sector and other stakeholders can develop policies and programs to help control smoking among schoolchildren.

Approximately, one-third of smoker schoolchildren (38.2%) had intention to quit smoking in the present study. This is in contrast to the results of a study of schoolchildren in Madinah, Saudi Arabia, where that 71.7% of smoker schoolchildren had the intention to quit [22]. Similarly, another study in Jeddah, Saudi Arabia, reported a considerably high percentage of smoker schoolchildren (63.2%) having the intention to quit smoking [14]. Kentala et al. followed adolescents for two years who received educational intervention based on oral hygiene and found that 73% of the participants in the intervention group compared with 63% of controls claimed to quit smoking [28]. However, a low quitting ratio of less than 20% was found in China, India, Russia, Bangladesh, and Egypt, and it was found that relatively fewer smokers will quit smoking if current prevalence rates of smoking and cessation patterns persist worldwide [29]. A possible reason for the variations in the reporting of the intention of quitting in different studies could be because of discrepancies in defining the intention to quit smoking. In accordance with a previous study, the intention to quit smoking in our study was defined as quitting within the next six months [30]. Furthermore, a low prevalence of intention of quitting in our study could be related to the lack of smoking awareness campaigns and programs among schools in the Eastern province of Saudi Arabia.

The literature reports an increased risk of periodontal disease, dental caries, tooth sensitivity, and oral cancer with tobacco use [28, 31]. Our study also found tooth sensitivity, dental cavities, and dental pain highly prevalent among smokers as about half of them reported having these dental problems. Moreover, these findings are consistent with the results of a previous study that observed bleeding gums, tooth sensitivity, and dental cavities in almost half the sample of 10–12 grade schoolchildren in the Eastern province of Saudi Arabia [11].

The logistic regression final model in our study revealed that schoolchildren who started smoking within the last two years were almost four times (OR = 3.97) more likely to quit smoking than those who initiated smoking for more than two years ago. In agreement with the literature, the increasing age is associated with reduced intention of quitting [32]. The individuals who started smoking at a younger age (before 16 years) were twice more likely not to quit smoking than those who began smoking at a later age [33]. It is possible that the participants who started tobacco consumption within the initial few years may have a lower level of nicotine dependence and hence may show increased intention to quit smoking [24]. Another explanation could be that the schoolchildren who have been smoking for a longer period may be under the greater influence of smoker family members, which can hinder their quitting intention [34]. In the present study, about three-quarters of participants had a family history of smoking.

Smokers with high education have increased chances of expressing their intention to quitting than those with low education [30, 32]. Our data indicate the positive impact of higher education of mothers on the intention of quitting among schoolchildren. The present study found that the children of mothers with university education were 2.76 times more likely to have the intention to quit than the children of school-educated mothers. Therefore, encouraging higher education among mothers might be a good addition to smoking prevention and abstinence initiatives.

The influence of the perception about the health hazards of smoking on the intention of quitting is known. The literature shows that tobacco-related health concern is the most common reason for smoking cessation [15]. It was found that the subjects who perceived adverse consequences of tobacco on health were more likely to express the intention to quit smoking [22, 34]. Similarly, adolescents reported health as the most common reason for expressing the willingness to quit smoking [35]. A study reported that when smokers were diagnosed with tuberculosis, 23.8% of them quitted smoking, while out of the remaining 76.2% of smokers, 52.4% intended to quit [20]. In our study, tooth sensitivity and dental pain were significant predictors of quitting smoking in schoolchildren. Moreover, schoolchildren with tooth sensitivity were 3.2 times more likely than those without tooth sensitivity to join a smoking cessation program.

The present study provides a unique opportunity to analyze oral health-related predictors of quitting smoking among schoolchildren. However, certain limitations of the study should be considered. One of the limitations was the recruitment of only male schoolchildren in the study. This is because the data collection from female schools could be challenging for male researchers due to administrative, social, and cultural difficulties in the country. The study used self-reported data on dental cavities, although oral examination provides more accurate information about dental caries. There is also the possibility of underreporting and overreporting of responses that can lead to bias in survey research. The future study should consider randomized controlled design to evaluate if schoolchildren with tooth sensitivity and dental pain actually quit smoking.

## 5. Conclusions

The study found that a clear majority of schoolchildren had a family history of smoking, and half of the participants started smoking within the last two years. Tooth sensitivity, dental cavities, and dental pain were highly prevalent among smokers. Almost one-quarter of smokers had the intention to quit smoking and join a smoking cessation program. The initiation of smoking within two years, dental pain, tooth sensitivity, and having university-educated mother were significantly and independently associated with high odds of quitting smoking. The students with tooth sensitivity were more likely to join a smoking cessation program than those without tooth sensitivity.

Health policymakers and stakeholders should consider the role of family history of smoking and university education of mothers when developing measures and initiatives to prevent and control smoking in schoolchildren. The schoolchildren who recently started smoking should be the target of tobacco control programs. Health care professionals should promote tobacco prevention among smokers who are diagnosed with tooth sensitivity and or dental pain and refer them to a smoking cessation program because they are more likely to quit smoking. The awareness about the adverse effects of smoking on oral health should be part of regional, national, and global tobacco prevention policies and programs.

## Figures and Tables

**Table 1:** Descriptive analysis: frequency distribution of study variables.

Variables	Frequency/percentage (*N*/%)
Academic grades	
More than 80%	153 (76.9)
Less than 80%	46 (23.1)
Family income	
≥SR 10000 (equal to US$ 3733)	136 (68.3)
<SR 10000 (equal to US$ 3733)	63 (31.7)
Father's education	
≥College/University education	119 (59.8)
< School education	80 (40.2)
Mother's education	
≥College/University education	104 (52.3)
< School education	95 (47.7)
Initiation of smoking	
≤2 years	103 (51.8)
>2 years	96 (48.2)
Family history of smoking	151 (75.9)
Routine dental check-up	43 (21.6)
Bleeding of gums in the past month	62 (31.2)
Dental cavities	52.8 (52.8)
Dental pain in the past month	89 (44.7)
Tooth sensitivity in the past month	112 (56.3)
Dryness of the mouth	62 (31.2)
Malodor	59 (29.6)
Satisfaction with appearance	98 (49.2)
Intention to quit smoking	76 (38.2)
Intention to join smoking cessation program	67 (33.7)

**Table 2:** Factors associated with the intention to quit smoking.

Variables	Unadjusted OR (95% CI)	*P* value	Adjusted OR (95% CI)	*P* value
Academic grades				
More than 80%	1.78 (0.87, 3.64)	0.114	1.86 (0.58, 5.96)	0.298
Less than 80%				
Family income				
≥SR 10000 (equal to US$ 3733)	1.11 (0.6, 2.06)	0.739	2.2 (0.87, 5.54)	0.093
<SR 10000 (equal to US$ 3733)				
Father's education				
≥College/University education	1.45 (0.69, 3.02)	0.320	0.65 (0.18, 2.31)	0.509
< School education				
Mother's education				
≥College/University education	2.1 (1.01, 4.33)	0.044^*∗*^	2.63 (0.75, 9.21)	0.129
< School education				
Initiation of smoking				
≤2 years	2.54 (1.4, 4.6)	0.002^*∗*^	3.69 (1.46, 9.31)	0.006^*∗*^
>2 years				
Family history of smoking	1.17 (0.59, 2.3)	0.650	0.61 (0.21, 1.73)	0.350
Routine dental check-up	0.95 (0.47, 1.9)	0.881	1.87 (0.63, 5.51)	0.256
Bleeding of gums in the past month	1.03 (0.56, 1.91)	0.919	0.64 (0.24, 1.68)	0.362
Dental cavities	1.17 (0.66, 2.09)	0.579	0.76 (0.3, 1.89)	0.559
Dental pain in the past month	2.61 (1.45, 4.69)	0.001^*∗*^	3.29 (1.31, 8.25)	0.011^*∗*^
Tooth sensitivity in the past month	2.27 (1.25, 4.14)	0.007^*∗*^	4.16 (1.48, 11.70)	0.007^*∗*^
Dryness of the mouth	1.38 (0.75, 2.55)	0.295	0.87 (0.34, 2.26)	0.779
Malodor	2.11 (1.14, 3.93)	0.017	1.24 (0.47, 3.27)	0.669
Satisfaction with appearance	0.68 (0.38, 1.22)	0.196	0.63 (0.27, 1.49)	0.293

^*∗*^Statistically significant.

**Table 3:** Multivariate logistic final model (backward LR): factors associated intention to quit smoking.

Variables	Intention to quit smoking	
	Adjusted OR (95% CI)	*P* value
Dental pain in the past month	2.84 (1.24, 6.51)	0.014
Tooth sensitivity in the past month	3.7 (1.51, 9.06)	0.004
Initiation of smoking		
≤2 years	3.97 (1.68, 9.34)	0.002
>2 years		
Mother's education		
≥Bachelor's degree	2.76 (1.05, 7.29)	0.040
<Bachelor's degree		
Family income		
≥SR 10000 (equal to US$ 3733)	2.11 (0.91, 4.89)	0.083
<SR 10000 (equal to US$ 3733)		

^*∗*^Statistically significant.

**Table 4:** Factors associated with the intention to join a smoking cessation program.

Variables	Unadjusted OR (95% CI)	*P* value	Adjusted OR (95% CI)	*P* value
Academic grades				
More than 80%	0.73 (0.37, 1.45)	0.371	0.44 (0.14, 1.33)	0.145
Less than 80%				
Family income				
≥SR 10000 (equal to US$ 3733)	0.75 (0.40, 1.40)	0.368	0.69 (0.29, 1.62)	0.397
<SR 10000 (equal to US$ 3733)				
Father's education				
≥College/University education	0.87 (0.42, 1.83)	0.724	0.48 (0.14, 1.61)	0.234
<School education				
Mother's education				
≥College/University education	1.5 (0.73, 3.08)	0.269	3.55 (0.99, 12.73)	0.052
<School education				
Initiation of smoking				
≤2 years	1.03 (0.57, 1.85)	0.923	1.14 (0.5, 2.61)	0.752
>2 years				
Family history of smoking	1.16 (0.57, 2.32)	0.684	0.84 (0.3, 2.34)	0.734
Routine dental check-up	1.22 (0.6, 2.46)	0.579	2.26 (0.77, 6.67)	0.139
Bleeding of gums in the past month	1.69 (0.91, 3.15)	0.097	1.09 (0.43, 2.76)	0.858
Dental cavities	1.39 (0.77, 2.52)	0.273	0.83 (0.34, 2.02)	0.686
Dental pain in the past month	2.28 (1.25, 4.16)	0.006^*∗*^	1.88 (0.78, 4.53)	0.158
Tooth sensitivity in the past month	3.32 (1.74, 6.35)	<0.001^*∗*^	3.02 (1.14, 7.96)	0.026^*∗*^
Dryness of the mouth	1.38 (0.74, 2.58)	0.311	0.67 (0.26, 1.73)	0.410
Malodor	1.13 (0.6, 2.14)	0.709	1.27 (0.49, 3.32)	0.621
Satisfaction with appearance	1.44 (0.79, 2.59)	0.229	1.67 (0.72, 3.88)	0.229

^*∗*^Statistically significant.

**Table 5:** Multivariate logistic final model (backward LR): factors associated with the intention to join a smoking cessation program.

Variables	Intention to join a smoking cessation program	
	Adjusted OR (95% CI)	*P* value
Dental pain in the past month	2.05 (0.94, 4.47)	0.071
Tooth sensitivity in the past month	3.22 (1.37, 7.53)	0.007^*∗*^
Routine dental check-up	2.29 (0.85, 6.17)	0.100

^*∗*^Statistically significant.

## Data Availability

The SPSS data file of this study is available from the corresponding author upon request.

## Supplementary Materials

Supplementary Materials The supplementary material is a questionnaire or instrument, which was submitted through journal's online submission system. The description of this instrument or questionnaire is mentioned in the manuscript.
