# Supplementary material for: Influence of Dental Pain and Tooth Sensitivity on the Intention to Quit Smoking among Schoolchildren
Source: Int J Dent. 2020 Jul 9;2020:8823146. doi: 10.1155/2020/8823146 (PMC7368192; doi:10.1155/2020/8823146)
Supplement: Supplementary Materials — The supplementary material is a questionnaire or instrument, which was submitted through journal's online submission system. The description of this instrument or questionnaire is mentioned in the manuscript. [file 8823146.f1.docx]

**QUESTIONNAIRE**

**Sociodemographic Information**

- **Age: ---------years**
- **School level :**

□ 10^th^ Grade

□ 11^th^ Grade

□ 12^th^ Grade

- **Academic grade in previous year?**

□ Less than 80%

□ Equal or more than 80%

- **Monthly family income?**

□ Less than SR 10000 (Equal to US$ 3733)

□ Equal or more than SR 10000 (Equal to US$ 3733)

- **Father’s educational level?**

□ School education

□ College/university education

- **Mother’s educational level?**

□ School education

□ College/university education

Q 1. Are you a current smoker?

□ Yes

□ No

Q 2. If yes, when did you initiate smoking?

□ Equal or less than 2 years

□ More than 2 years

Q 3. Do you have a family history of smoking?

□ YES

□ NO

**Oral Health Problems**

Q 1. Do you visit your dentist for a routine check-up?

□ YES

□ NO

Q 2. Did you have bleeding gum in the past month?

□ YES

□ NO

Q 3. Do you have cavities in your teeth?

□ YES

□ NO

Q 4. Did you experience pain in your teeth in the past month?

□ YES

□ NO

Q 5. Did you experience tooth sensitivity to cold / hot in the past month?

□ YES

□ NO

Q 6. Do you have dryness of mouth?

□ YES

□ NO

Q 7. Do you have bad breath?

□ YES

□ NO

Q 8. Are you satisfied with the appearance of your teeth?

□ YES

□ NO

**Intention to quit smoking and join a smoking cessation program**

Q 1. Do you intend to quit smoking in the next six months?

□ Yes

□ No

Q 2. If yes, do you intend to join a smoking cessation program?

□ Yes

□ No

**Suggestions**

**--------------------------------------------------------------------------------------------------------------------------------------------------------------------------------------------------------------------------------------------------------------------------------------------------------------------------------------------------------------------------------------------------------------------------------------------------------------------------------------**

**Thank you for your participation in the study**
